# Manipulation under Anaesthesia for Patient Reported Stiffness after Total Knee Arthroplasty in an Asian Population

**DOI:** 10.5704/MOJ.2003.009

**Published:** 2020-03

**Authors:** HC Boo, SJ Yeo, HC Chong

**Affiliations:** Department of Orthopaedic Surgery, Singapore General Hospital, Singapore

**Keywords:** knee arthroplasty, functional outcomes, knee stiffness, manipulation under anaesthesia

## Abstract

**Introduction:**

Stiffness after Total Knee Arthroplasty (TKA) is a complication that decreases patient satisfaction. Patients in an Asian population have potentially different requirements of knee range of motion. The authors have encountered patients who complain of subjective stiffness post TKA who do not have a severely restricting range of motion (ROM). Some patients have persistent subjective stiffness and undergone Manipulation Under Anaesthesia (MUA). We look at their functional outcomes post MUA.

**Materials and Methods:**

This is a retrospective study, including 48 patients from a single institution who underwent MUA for stiffness, separated into objective and subjective knee stiffness. Patients with subjective knee stiffness who underwent MUA had failed conservative management. ROM, Oxford Knee Scores (OKS), Knee Society Scores (KSS) and Short Form 36 (SF36) scores were compared at two years post MUA.

**Results:**

The demographics of the two patient groups were similar. The time interval between index TKA and MUA was higher in the subjective knee stiffness group. Pre-MUA OKS, KS Function Score, KSS and SF36 scores were similar in both patient subgroups. There was no significant difference in the OKS, KSS or SF36 at two year follow-up. The proportion of patients in each group who achieved the Minimum Clinically Important Difference (MCID) improvement in the scores was also similar.

**Conclusions:**

Patients with subjective knee stiffness can achieve similar functional outcome improvements in Oxford and Knee Society Scores with MUA at two years follow-up.

## Introduction

Stiffness after Total Knee Arthroplasty (TKA) is a known and frustrating complication that decreases patient satisfaction and adversely affects surgical outcomes. The definition of knee stiffness is variable, with objective measurements described by Kim *et al*^1^ as flexion contracture of > 15º and/or < 75º of flexion of the knee, and Christensen *et al*^2^ describing it as an arc of knee motion < 70º, or subjective such as limited Range of Movement (ROM) affecting a patient’s ability in performing daily activities of living^3^.

Numerous causes have been postulated as risk factors for post-operative stiffness, of which pre-operative stiffness is significant^4^. Other patient factors include diabetes, smoking, and lung disease^5-7^. Management of stiffness can be challenging with varied guidelines and options. Various treatment options are available for the management of stiffness after TKA. Patients are routinely put through aggressive physiotherapy, and should physiotherapy fail to help the patient obtain a functional ROM, procedural intervention may be considered. One of the available and proven options includes Manipulation Under Anaesthesia (MUA) which has been shown to improve ROM without patients undergoing revision surgery^8,9^.

The range of motion attained post TKA directly affects knee function as well as patient satisfaction scores. Literature has identified the gross definition of knee stiffness, but these studies are largely conducted in the western populations. In the Asian population, varying demands for knee ROM have been studied. Mamarelis *et al*^10^ described the variation in flexion requirements due to regional differences, with certain population groups requiring more than 130º flexion for specific activities such as kneeling for prayers or sitting cross-legged.

The authors have encountered patients with poor satisfaction post-operatively after TKA due to complaints of subjective knee stiffness, which did not fulfill the commonly accepted criteria of knee stiffness. Some of these patients subsequently underwent MUA for subjective knee stiffness, after having failed conservative therapy with physical rehabilitation. The authors looked at this group of patients to evaluate if they had significant improvements in functional outcome scores and satisfaction post MUA.

## Materials and Methods

Institutional review board approval (Ref. No. 20150200147) was obtained for completion of this retrospective cohort study. We reviewed patient data for patients who underwent MUA for stiffness post TKA from our institution between January 2004 and December 2014.

A total of 48 patients (mean age 63.1 (38 to 79)) who underwent MUA for knee stiffness following TKA were identified from our single centre database and followed up for two years, out of a total of approximately 10000 total knee arthroplasties performed over the same period. Patients with objective indications for MUA include objective poor ROM < 70º, flexion contracture >15º, poor maximal flexion <90º. Subjective indications for MUA included subjective self-reported difficulty in performing activities (e.g. kneeling for prayers) of living due to stiffness in patients whom physiotherapy rehabilitation have failed to produce improvement in symptoms over a minimal period of three months.

For the purpose of the study, revision TKA cases with generally poorer outcomes compared to primary TKAs were excluded. All primary TKAs were done by consultant orthopaedic surgeons for primary osteoarthritis. None of the patients in the study population suffered from rheumatological conditions or suffered from peri-operative complications (e.g. periprosthetic fractures, early deep surgical infections, deep vein thrombosis) for the primary TKA that could have adversely affected their clinical outcomes. After the index TKA, patients were allowed full weight bearing status post-operative day one, and were discharged when they were able to ambulate and climb a single flight of stairs of four steps with minimal assistance via ambulatory aids.

Post-MUA routine clinic visits were scheduled at regular intervals at 1 month, 3 months, 6 months, 12 months and 24 months and functional scores were captured at the 6 months and 24 months visits. Patients continued to undergo a physiotherapy rehabilitation programme post-MUA. Range of motion was assessed at the one month follow-up and physiotherapy reinforced if they were unable to attain 90º knee flexion. Patients who reported subjective knee stiffness were encouraged to undergo a longer duration of physiotherapy.

In patients with subjective knee stiffness, MUA was only performed in patients who felt persistent knee stiffness adversely affecting satisfaction rates and which did not improve despite a longer trial of physiotherapy. Post MUA rehabilitation protocols were similar to the index TKA, with additional effort made to obtain knee flexion of 90º before discharge. Physiotherapy was commenced on day one post-MUA with full weight bearing physiotherapy. Additional focus was placed on improving knee ROM whilst inpatient. Patients were discharged when deemed competent to ambulate safely with the use of walking aids, as well as climb at least 4 steps of stairs. Follow-up visits were scheduled at closer intervals and physiotherapy was strictly continued in the outpatient setting to reinforce compliance and assess patient progress.

Patient data including ROM, Knee Society Scores^11^, Oxford Knee Scores, Knee Society Function Scores^12^ and SF-36 scores were collected at the Orthopaedic Diagnostic Centre (ODC) from our hospital during follow-up consultations with the operating surgeon.

Patient data was obtained from standardised case notes and electronic records for the purpose of this study. MUA was performed under General Anaesthesia for all patients. The ipsilateral hip was flexed to 90º and steady loading was applied progressively to the knee till palpable break of adhesions were detected. No further force was applied beyond this firm point.

Statistical analysis was performed using the Statistical Package for the Social Sciences (SPSS v.20). Categorical data was analysed using chi-squared or Fisher’s exact tests, whilst other data were analysed using paired t- tests. A p-value of <0.05 was interpreted as significant.

## Results

The study included 48 patients, including 19 patients with objective knee stiffness, and 29 patients with subjective knee stiffness. The demographics of the patient groups were similar in terms of age, sex and BMI (Table I). The interval between index TKA and MUA was significantly longer in the group with subjective knee stiffness as expected, due to a longer trial of conservative management before intervention.

**Table I:** Demographic comparison between Objective and Subjective Knee Stiffness Groups

	Objective (σ)	Subjective (σ)	P Value
Age	60.3 (9.4)	65.9 (8.6)	0.05
Pre-MUA Body Mass Index (BMI)	29.4 (4.5)	27.7 (4.8)	0.25
Sex	Male = 2	Male = 4	0.74
	Female = 17	Female = 25	
Interval between MUA and Index TKA (in months)	3.3 (7.9)	10.3 (17.7)	0.10

Pre-MUA mean ROM was statistically different, with a mean ROM of 62º in the objective and 109º (P < 0.01) in the subjective knee stiffness group. Functional scores obtained pre-MUA were not statistically different between the two groups pre-MUA. The SF36 scores, broken into component sections, did not show any significant difference (Table II). Post-MUA ROM was statistically different in the objective knee stiffness group at 83º and 105º in the subjective knee stiffness group (P < 0.01). However, the functional knee scores and their respective mean score improvements were not statistically different. The mean improvement in Oxford Knee Scores of 15.5 and 16.0 (p=0.85), Knee Society scores of 50.0 and 38.5 (P = 0.13) and Knee Society Function Scores of 24.5 and 20.5 (p=0.49) in the objective and subjective knee stiffness groups were not statistically different. SF36 component scores were not statistically different at two years post-MUA (Table III).

**Table II:** Pre-Mua Comparison of Knee Rom, Sf36 and Functional Outcomes Scores between Objective and Subjective Knee

	Objective (σ)	Subjective (σ)	P Value
Pre-MUA ROM (degrees)	62° (27.7)	109° (16.7)	< 0.01 (47)
Oxford Knee Scores	35.3 (6.8)	37.6 (8.6)	0.32
Knee Society Function Scores	52.9 (18.8)	52.7 (21.0)	0.98
Knee Society Scores	30.4 (19.2)	40.8 13.6)	0.06
SF36 - Physical Function	37.1	40.0	0.67
SF36 - Role Function (Physical)	16.7	25.0	0.41
SF36 - Bodily Pain	34.5	32.4	0.67
SF36 - General Health	72.7	65.7	0.71
SF36 - Vitality	62.4	59.4	0.27
SF36- Social Function	57.1	52.3	0.66
SF36- Role Function (emotional)	79.4	80.2	0.94
SF36 - Mental Health	66.5	73.9	0.32

**Table III:** Comparison of Rom and Knee Scores at two years between Objective and Subjective Knee Stiffness Groups

	Objective (σ)	Subjective (σ)	P Value
Knee ROM	83° (19.4)	105° (18.5)	< 0.01 (22)
Oxford Knee Scores	19.8 (5.4)	21.6 (6.7)	0.34
Knee Society Scores	77.4 (11.4)	73.3 (19.3)	0.43
Knee Society Function Scores	80.4 (12.9)	80.4 (19.3)	0.99
SF36 - Physical Function	69.5	65.0	0.40
SF36 - Role Function (Physical)	72.6	72.2	0.97
SF36 - Bodily Pain	63.9	64.7	0.91
SF36 - General Health	70.6	71.0	0.94
SF36 - Vitality	70.2	68.0	0.77
SF36- Social Function	86.3	88.0	0.82
SF36- Role Function (emotional)	79.4	86.4	0.51
SF36 - Mental Health	78.7	83.3	0.37
Oxford Knee Scores mean improvement	15.5	16.0	0.85
Knee Society Score mean improvement	50.0	38.5	0.13
Knee Society Function Score mean improvement	24.5	20.5	0.49

Patients with objective knee stiffness experienced an average improvement in knee ROM of 24º, whilst the subjective knee group did not experience significant change in the knee ROM (Table IV). The proportion of patients in each group who achieved Minimal Clinical Important Difference (MCID) improvement in the knee functional outcome scores were not statistically different (Table V).

**Table IV:** Comparison of RRom Pre and Post MUA

	Pre MUA Knee ROM (σ)	Post MUA Knee ROM (σ)	P Value
Objective	62° (26.3)	109° (15.0)	< 0.01
Subjective	109°	105°	0.33

**Table V:** SF36 Component Score Comparison Pre-MUA and six months, two years Post MUA.

	Pre – MUA	Six months post MUA	P Value (six months	Two years post MUA	P Value (Two years post MUA)
Physical Function	Obj: 37.1	Obj: 62.3	P = 0.00	Obj: 69.5	P < 0.01
	Subj: 40.0	Subj: 63.6	P = 0.00	Subj: 65.0	P < 0.01
Role Function (Physical)	Obj: 16.7	Obj: 72.1	P = 0.00	Obj: 72.6	P < 0.01
	Subj: 25.0	Subj: 60.7	P = 0.02	Subj: 72.2	P < 0.01
Bodily Pain	Obj: 34.5	Obj: 70.9	P = 0.00	Obj: 63.9	P < 0.01
	Subj: 32.4	Subj: 58.3	P = 0.00	Subj: 64.7	P < 0.01
General Health	Obj: 72.7	Obj: 75.1	P = 0.33	Obj: 70.6	P = 0.54
	Subj: 65.7	Subj: 69.2	P = 0.60	Subj: 71.0	P = 0.28
Vitality	Obj: 62.4	Obj: 66.5	P = 0.62	Obj: 70.2	P = 0.18
	Subj: 59.4	Subj: 71.0	P = 0.96	Subj: 68.0	P = 0.14
Social Function	Obj: 57.1	Obj: 79.4	P = 0.00	Obj: 86.3	P < 0.01
	Subj: 52.3	Subj: 75.6	P = 0.22	Subj: 88.0	P < 0.01
Role Function (emotional)	Obj: 79.4	Obj: 78.4	P = 0.83	Obj: 79.4	P = 1.00
	Subj: 80.2	Subj: 88.9	P = 0.34	Subj: 86.4	P = 0.36
Mental Health	Obj: 66.5	Obj: 75.8	P = 0.14	Obj: 78.7	P = 0.03
	Subj: 73.9	Subj: 80.6	P = 0.18	Subj: 83.3	P = 0.48

SF36 scores at the six month and two year time points were compared to the pre-MUA scores. There were statistically significant improvements in component scores for the sections of physical function, bodily pain and social function in both groups (Table VI). Notably, the mental health component score improved significantly for the objective knee stiffness group, but not the subjective knee stiffness group.

## Discussion

Stiffness post TKA remains an important clinical complication. Clinicians will encounter patients with stiffness who require intervention. However, there may also exist a group of patients who experience poor satisfaction due to subjective knee stiffness. There is a dilemma on the management of such patients, as they have good knee ROM on objective measurements, but remain unsatisfied with their surgical outcomes.

In our Asian population, this occurrence may be more common due to the varying expectations of ROM post-TKA. The attending surgeon is inclined to persist with conservative management for patients with subjective knee stiffness before considering any intervention given the lack of evidence supporting MUA^11^ for such cases.

Our results reflect this management algorithm as the time interval between the index TKA and MUA is significantly higher in the subjective knee stiffness group, due to a longer trial of physiotherapy before considering intervention. Our results suggest that such cases do benefit from MUA with improved functional outcome scores post-MUA.

In the comparison of outcome scores in both patient groups, we note that the functional outcome scores pre MUA were similar. The absolute increase, as well as the functional outcome scores at two years post MUA were also similar. Both groups showed significant increase in both the Knee Society Score as well as the Knee Society Function Score at two years post MUA. The proportion of patients in each subgroup who achieved the MCID improvement for the Knee Society Scores were also similar between the two patient groups. This suggests that patients in the subjective knee stiffness group did experience a statistically significant improvement in functional outcome scores post-MUA.

There were also significant improvements in the SF36 component scores such as physical function, perceived emotional and physical improvements in abilities to participate in daily activities, as well as overall pain

The authors note that there was a significant improvement in pain and social function component scores in patients with subjective knee stiffness. This may account for the improvement in their functional outcome scores at two years despite not having a demonstrable improvement in ROM. Although the mechanism is not apparent, the improvement in scores do translate into clinically significant difference. The authors hypothesise that psychological factors may play a role, although further research in this aspect is required.

Overall, the results suggest that patients experience similar improvement in functional outcome scores up to two years post MUA. In this select group of patients who present with subjective knee stiffness post TKA which has failed a trial of physiotherapy, and who score poorly on validated objective questionnaires, MUA may have a role in improving patient satisfaction.

Several complications from MUA have been reported in the literature, such as fractures^13^, wound dehiscence, patellar ligament avulsions, haemarthrosis, heterotopic bone formation and pulmonary embolism^14-17^. These complications are generally more commonly noticed in late manipulations. There were no similar reported complications associated with MUA in this study. The authors hypothesise that given the acceptable pre-MUA ROM in the subjective knee stiffness group, the surgeons performing MUA are less likely to be overly aggressive with the MUA and therefore are less likely to experience complications.

Overall, our findings are in agreement with available literature which suggests that MUA provides significant improvement in ROM^18^ in patients who experience objective knee stiffness. For patients presenting with subjective knee stiffness, our results demonstrate comparable improvement in functional knee scores at two years despite a lack of quantifiable improvement in ROM.

Looking at these results, the authors hypothesise that subjective factors such as patient expectations may play a larger role in post TKA satisfaction rates than we expect, and poor patient satisfaction from stiffness may not be solely based on an isolated measurement of ROM. Mental factors may be contributory to the perception of stiffness. It is possible that MUA may contribute to a decreased perception of terminal stiffness, thus achieving the intended perception of decreased stiffness, despite not having objective improvements in ROM in this select group of patients. These subjective effects which have been studied in literature^19,20^, may not be easily quantifiable by objective measurements. This may explain why the improvement in and the eventual functional outcome scores are similar between the two patient groups. Functional outcome questionnaires may be useful to identify patients in this group with low SF-36 component scores who may benefit from MUA.

The authors also note the similar improvement made in both patient groups, despite the subjective knee stiffness group having a higher delay between the index TKA and MUA. This suggests timing is less critical in this select group of patients. The optimal timing for MUA is still debatable, with literature supporting both early^11^ and late^21^ intervention. The clinician is more likely to persist with non-invasive treatment modalities for a longer period of time with patients with subjective knee stiffness. The decision for this treatment plan however, does not appear to compromise the outcomes should the clinician decide to perform an MUA at a later stage.

A limitation of this study includes the small study population. Further studies looking at the correlation between specific functional outcome component scores and patient satisfaction may substantiate the findings here. More clinical studies may also be required to investigate the impact of subjective factors such as patient expectations.

## Conclusion

Knee Stiffness post TKA is a challenging and frustrating outcome for both patient and surgeon. MUA is considered as an initial non-surgical option that is useful in improving ROM in managing objective knee stiffness^22,23^.

In patients who present with persistent subjective knee stiffness post TKA that was functionally limiting, clinically significant improvement in functional outcome scores was achieved with MUA if the patient had undergo a trial of physiotherapy with no symptomatic improvement. Pre-MUA functional questionnaires can be administered to identify patients with low component scores who may benefit from MUA. For the clinician who is faced with this dilemma of performing intervention for patients with subjective knee stiffness there is time for a trial of conservative therapy, as MUA performed at a later stage did not seem to significantly affect the outcomes post MUA.
